# Genome-Wide Analysis of *DREB* Genes Identifies a Novel Salt Tolerance Gene in Wild Soybean (*Glycine soja*)

**DOI:** 10.3389/fpls.2022.821647

**Published:** 2022-03-04

**Authors:** Zhihong Hou, Yongli Li, Yuhan Cheng, Weiwei Li, Tai Li, Hao Du, Fanjiang Kong, Lidong Dong, Dianfeng Zheng, Naijie Feng, Baohui Liu, Qun Cheng

**Affiliations:** ^1^College of Agriculture, Heilongjiang Bayi Agricultural University, Daqing, China; ^2^Innovative Center of Molecular Genetics and Evolution, School of Life Sciences, Guangzhou University, Guangzhou, China; ^3^Beijing Zhongnong Futong Horticulture Co., Ltd., Beijing, China; ^4^Keshan Branch of Heilongjiang Academy of Agricultural Sciences, Keshan, China; ^5^College of Coastal Agricultural Sciences, Guangdong Ocean University, Zhanjiang, China; ^6^Shenzhen Research Institute of Guangdong Ocean University, Shenzhen, China

**Keywords:** artificial selection, DREB, natural variation, salt tolerance, cultivated soybean, wild soybean

## Abstract

Salt stress is a major factor limiting the growth and yield of soybean (*Glycine max*). Wild soybeans (*Glycine soja*) contain high allelic diversity and beneficial alleles that can be re-introduced into domesticated soybeans to improve adaption to the environment. However, very few beneficial alleles have been identified from wild soybean. Here, we demonstrate that wild soybean is more salt tolerant than cultivated soybean and examine dehydration responsive element-binding (DREB) family transcription factor genes to look for advantageous alleles that might improve drought tolerance in cultivated soybean. Our genome-wide analysis identified 103 *DREB* genes from the *Glycine max* genome. By combined RNA-sequencing and population genetics of wild, landrace, and cultivated soybean accessions, we show that the natural variation in *DREB3a* and *DREB3b* is related to differences in salt tolerance in soybean accessions. Interestingly, *DREB3b*, but not *DREB3a*, appears to have undergone artificial selection. Soybean plants carrying the wild soybean *DREB3b* allele (*DREB3b^39Del^*) are more salt tolerant than those containing the reference genome allele (*DREB3b^Ref^*). Together, our results suggest that the loss of the *DREB3b^39Del^* allele through domestication of cultivated soybean may be associated with a reduction in salt tolerance. Our findings provide crucial information for improving salt tolerance in soybean through molecular breeding.

## Introduction

High salt levels in soil cause stress that affects plant growth and salinization of farmland restricts global agricultural production (Flowers, 2004; Munns and Tester, 2008; Ondrasek et al., 2011). Soil salinization is exacerbated by natural and human activities, including irrigation (Munns et al., 2020). Under salt stress, plants experience dehydration, metabolic toxicity, nutrient deficiencies, and membrane dysfunction, leading to tissue damage and early senescence (Essah et al., 2003). To adapt to various environmental stresses, plants have evolved many complexes signaling networks, and the molecular basis of these pathways is currently being studied (Zhu, 2001).

Soybean [*Glycine max* (L.) Merr.] is an economically important crop for oil and protein (Graham and Vance, 2003). It is classified as a moderately salt-sensitive crop; under salt stress, soybean yields may be reduced by up to 40% (Papiernik et al., 2005; Munns and Tester, 2008). Moreover, soybean cultivars show variation in their salt sensitivity: under saline conditions, salt-sensitive cultivars have a 37% decrease in yield compared with salt-tolerant cultivars (Parker et al., 1983). This natural variation in salt tolerance provides opportunities to increase soybean production under saline conditions (Guan et al., 2014; Qi et al., 2014). However, few salt-tolerant variants have been identified (Guan et al., 2014; Qi et al., 2014). This lack of knowledge has greatly inhibited attempts to improve salt tolerance in crops.

Cultivated soybean was domesticated from its wild counterpart *Glycine soja* Sieb. & Zucc. 6,000–9,000 years ago (Hymowitz, 1970; Carter et al., 2004). Due to artificial selection and population/genetic bottlenecks, cultivated soybeans have much lower genetic diversity than their wild relative (Hyten et al., 2006; Lam et al., 2010). This reduced variation may have led to the loss of some genes or alleles that are important for adapting to different environments. Therefore, wild soybean, with its high allelic diversity, may be a good source for beneficial alleles that can be re-introduced into domesticated soybean cultivars through breeding.

The dehydration responsive element-binding (DREB) family is a subgroup of the APETALA2/ethylene-responsive factor (AP2/ERF) transcription factor (TF) superfamily; in multiple plants, *DREB* genes respond to a wide variety of abiotic stresses, such as drought, high salt, cold, and heat (Xu et al., 2011; Kudo et al., 2017; Ali and Hadi, 2018; Eckardt, 2019). Some DREBs are involved in the salt stress response in soybean (Gao et al., 2005; Li et al., 2005; Chen et al., 2007). However, the variation in the *DREB* family in wild soybean remains to be explored. To fill this gap, we combined genome-wide analysis, association studies, population genetics, and RNA-sequencing to identify candidate causal *DREB* genes for salt tolerance. Our results show that *DREB3b* contributes to salt tolerance and may have undergone natural and artificial selection. In addition, the *DREB3b^39Del^* allele from wild soybean improves salt tolerance compared to the *DREB3b*^Ref^** allele from cultivated soybean. The identification of these *DREB3b* alleles provides important information for improving the yield of soybean and other crops.

## Materials and Methods

### Plant Materials, Salt Treatment, and Primers

The soybean cultivar Williams 82 (W82) and 424 soybean varieties (Lu et al., 2020) were used in this study. 424 soybean accessions were grown in a greenhouse using vermiculite as the growth medium, and maintained at 25°C and 70% relative humidity with a 16 h light/8 h dark. Fifteen days after planting, seedlings at the first-node stage (V1; Fehr et al., 1971) were subjected to 200 mM NaCl treatment. After being treated for 2 weeks, salt tolerance index was determined with two independent experiments. Briefly, five to eight plants of each accession in each replication were scored for salt tolerance. A salt-tolerance rating for each of the accession was assigned by the respective level of leaf chlorosis (Liu et al., 2020). The salt-tolerance ratings ranged on a scale from 1 (normal green leaves) to 5 (complete death). All primers used for vector construction, PCR, and quantitative reverse transcription (qRT)-PCR assays for all target genes are listed in Supplementary Table 3.

### Identification and Bioinformatic Analysis of *DREB* Genes in Soybean Genome

The soybean genome in Phytozome^1^ was used to identify all members of *DREB* family genes. A database search was carried out against all the genome-coded soybean proteins with the BLASTP program (*E*-value < 1e–20 and coverage >75%) in TBtools (Chen et al., 2020) using 56 full-length amino acid sequences of DREB homologs from *A. thaliana* (Sakuma et al., 2002). Multiple alignment of the protein sequences of 159 *DREB* genes (56 from *A. thaliana* and 103 from soybean) were aligned by ClustalW. A neighbor-joining phylogenetic tree was generated using MEGA_X with bootstrap repeated 1,000 times. The expression data of 103 *DREB* genes in different tissues (leaf, stem, root, flower, seed, pod, and cotyledon) of soybean were obtained from RNA-seq database (Machado et al., 2020).^2^ Location of *DREB* genes was visualized using the soybean genome generic feature format (.gff) using TBtools.

### RNA Extraction, Transcriptome Sequencing, and Quantitative Reverse Transcription–PCR

For RNA-sequencing, the W82 seedlings were grown in a growth chamber maintained at 25°C and 70% relative humidity with a 16-h light/8-h dark. Fifteen days after planting, seedlings at the first-node stage (V1; Fehr et al., 1971) were subjected to 200 mM NaCl treatment. The control plants were treated with water. After being treated for 2 weeks, total RNA was isolated using TRIzol reagent kit (Invitrogen, Carlsbad, CA, United States). cDNA synthesis was conducted using an M-MLV reverse transcriptase kit (Takara, Dalian, China) according to the manufacturer’s protocol. The cDNA fragments were purified using a QiaQuick PCR extraction kit, and ligated to Illumina sequencing adapters. The ligation products were size-selected by agarose gel electrophoresis, PCR amplified, and sequenced using an Illumina HiSeq 2500 system by Gene *Denovo* Biotechnology Co. (Guangzhou, China).

### Haplotype Network Construction

The full-length coding sequence of *DREB3a* and *DREB3b* in 1295 soybean accessions were retrieved from the 1295 resequencing data (Lu et al., 2020). The resequencing data of 1295 accessions were deposited into the NCBI database under accession number PRJNA394629, and the GSA database in BIG Data Center under accession numbers PRJCA000205 and PRJCA001691. Only haplotypes found in ≥3 soybean accessions were recorded. A haplotype network was constructed based on polymorphic sites of the whole coding sequences of *DREB3a* and *DREB3b* using the Median-Joining method in the NETWORK version 4.6.1.2 software (Fluxus Technology Ltd., Sudbury, Suffolk, United Kingdom) with data preparation using DNASP version 5 (Librado and Rozas, 2009).

### Nucleotide Diversity of *DREB3a* and *DREB3b*

A 20-kb sequence set (Chr01:48411780.48452989 and Chr15:1392382…1433465) centered on the candidate genes (*DREB3a* and *DREB3b*) were obtained from the 1295 soybean accessions whole-genome dataset. Nucleotide diversity (π) of each accession was estimated using the script vcftools with parameters–window-pi 20000–window-pi-step 10000.

### Plasmid Construction and Soybean Hairy Root Transformation

The coding sequence of *DREB3b* was obtained from W82 and GDW013 (wild soybean variety) by KOD-Plus-Neo (TOYOBO) with the primer in Supplementary Table 3. The coding sequence of *DREB3b* was cloned into plant expression vector pCAMBIA3301 with gene-specific primers. Transgenic soybean hairy roots were generated by *Agrobacterium rhizogenes*-mediated transformation as described by Graham et al. (2007) and Kereszt et al. (2007) with some modifications. The cotyledons were cut into rough triangles and immediately placed in Petri dishes containing 0.6% agar medium to keep them moist. The cut surface was treated with 20 μl *A. rhizogenes* suspension. The dishes were sealed with Parafilm and placed in an incubator at 25°C. Transformed hairy roots were abundant along a callus ridge on the inoculated cotyledons after approximately 3 weeks. Overexpression of the target gene in transgenic hairy roots was tested via quantitative PCR (qPCR) and GUS staining.

### Molecular Marker Development

The sequences of the *DREB3b^39Del^* allele and *DREB3b^Ref^* allele were obtained by sequencing. Primers were designed using Primer Premier 5.0, with a product size < 200 bp. The InDel marker was developed on the basis of the variation in *DREB3b*. Supplementary Table 3 lists the InDel markers that were used in this study.

### Statistical Analyses

In this study, for 424 accessions phenotypic evaluation, at least 6 individual plants were analyzed. all values were presented as mean ± s.e.m. and numbers (*n*) of samples or replicates are indicated in figure legends. Data were analyzed with GraphPad Prism 8 (ver. 8.0.1). Significance levels of differences were calculated by one-tailed, two-sample Student’s *t*-tests or one-way ANOVA with GraphPad Prism 8 (ver. 8.0.1).

## Results

### Genetic Diversity and Salt Tolerance Index in Soybean Accessions

**FIGURE 1:**
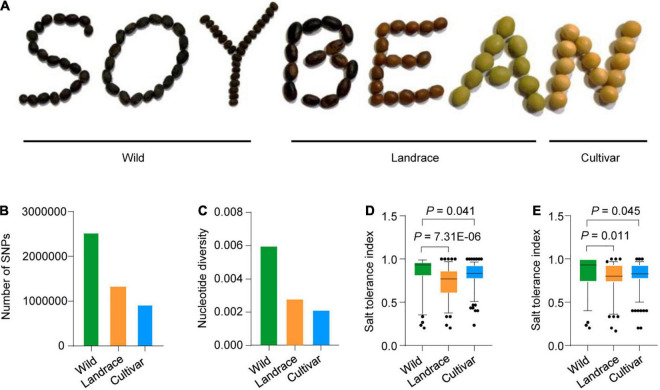
The morphologic characteristics and salt tolerance of wild, landrace and cultivated soybean. **(A)** The morphologic characteristics of wild, landrace, and cultivated soybean. **(B)** Number of SNPs of 424 accessions in chromosome 1 genomic region. **(C)** Nucleotide diversity of 424 accessions in chromosome 1 genomic region. **(D,E)** Comparison of the salt tolerance index of 424 accessions (85 wild soybeans, 153 landraces and 186 improved cultivars); two biological replicates in greenhouse.

Owing to “domestication syndrome” through artificial selection for traits to increase yield, cultivated soybean has lost substantial genetic diversity (Qiu et al., 2013; Li et al., 2014). Here, we examined a panel of 424 previously described accessions (Lu et al., 2020), which comprised 85 wild soybeans, 153 landraces, and 186 cultivars. To assess the genetic diversity in this panel, we compared the shapes of the wild, landrace, and cultivated soybean seeds (Figure 1A) and estimated the number of single-nucleotide polymorphisms (SNPs) and the nucleotide diversity across chromosome 1 in the 424 accessions. The number of SNPs and the nucleotide diversity are lowest in cultivated soybean and highest in wild soybean (Figures 1B,C). These results are consistent with a previous preliminary report (Lam et al., 2010).

We also measured the salt tolerance indexes of the 424-accession panel. Phenotype was evaluated in a growth chamber at 25°C, revealing significantly reduced salt tolerance in landrace and cultivated soybean compared to wild accessions in two biological replicates (Figures 1D,E). These results suggest that artificial selection may have had a strong effect on the genetic diversity in the cultivated soybeans.

### Genome-Wide Identification and Annotation of the *DREB* Gene Family

**FIGURE 2:**
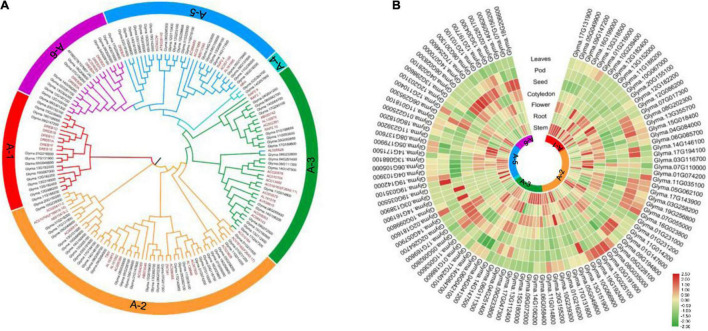
Phylogenetic tree of the *DREB* genes of *Arabidopsis thaliana* and soybean, and gene expression profile of soybean *DREB* genes in different tissues. **(A)** The NJ tree was constructed using MEGA_X based on alignment of a total of 159 DREB proteins, including 56 *Arabidopsis* DREBs and 103 *Glycine max* DREBs. DREB subfamilies are divided into six groups (A-1 to A-6). **(B)** The normalized microarray expression data of 103 *Glycine max* DREBs was downloaded from PLEXdb (http://www.plexdb.org/). The heatmap was produced using TBtools.

The DREB family plays an important role in plant responses to abiotic stresses (Gao et al., 2005; Chen et al., 2007; Ali and Hadi, 2018; Eckardt, 2019), so it would be meaningful to identify the genes encoding DREB TFs in soybean. In this study, 56 *Arabidopsis thaliana DREB* sequences (Sakuma et al., 2002) were used as a query to search the soybean database. This identified 103 *DREB* genes from the *Glycine max* genome (Supplementary Table 1), 73 of which have been reported previously (Zhou et al., 2020b). The *DREB* genes were distributed on all chromosomes of soybean, with 8, 3, 4, 5, 6, 8, 4, 3, 4, 5, 7, 7, 8, 9, 3, 2, 8, 2, 5, and 2 *DREB* genes on chromosome 1, 2, 3, 4, 5, 6, 7, 8, 9, 10, 11, 12, 13, 14, 15, 16, 17, 18, 19, and 20, respectively (Supplementary Figure 1). Phylogenetic analyses based on previously reported Arabidopsis *DREB* sequences (Sakuma et al., 2002) classified these genes into six subgroups: 14 genes in A-1 group, 35 genes in A-2 group, 19 genes in A-3 group, 2 genes in A-4 group, 21 genes in A-5 group, and 12 genes in A-6 group (Figure 2A and Supplementary Table 1).

### Expression Analysis of *DREB* Genes in Different Tissues

The transcript levels of 103 *DREB* genes in different soybean tissues were analyzed using the Phytozome database.^3^ The A-1 subgroup was highly expressed in stem and flower; the A-2 subgroup was highly expressed in root, flower, pod, and leaves; the A-3 subgroup was highly expressed in stem, root, and flower; the A-4 subgroup and the A-6 subgroup were highly expressed in seed and cotyledon; the A-5 subgroup was highly expressed in stem (Figure 2B). These data suggest that *DREB* genes of the same subgroup may have similar functions, whereas *DREB* genes of different subgroups may have functional differentiation.

### Expression Analysis of *DREB* Genes Under Salinity Stress

**FIGURE 3:**
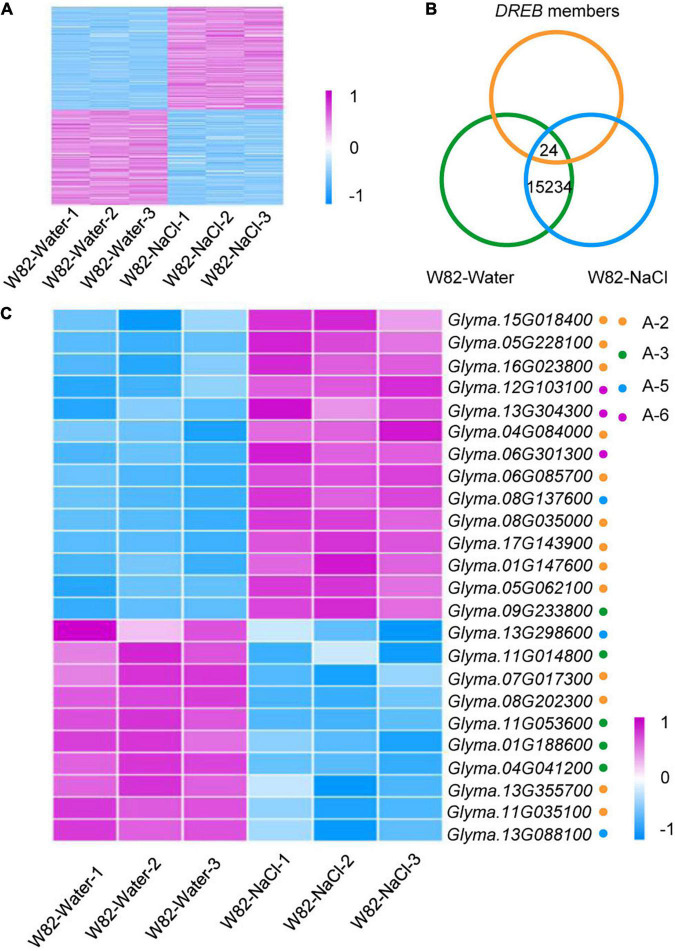
Identification of differentially expressed *DREB* genes in NaCl-treated W82 vs. mock treated. **(A)** Heat map of differentially expressed genes in W82-Water and W82-NaCl; *n* = 3 for each group. Relative transcript level is indicated on a color scale from purple (high) to blue (low). **(B)** Overlap between W82-Water, W82-NaCl and *DREB* genes. **(C)** Heat map of differentially expressed 24 *DREB* genes under NaCl treatment.

As a few *DREB* genes have been shown to be induced by salinity stress (Chen et al., 2007, 2009; Zhou et al., 2020a), we asked whether other *DREB* family members also control salt tolerance and whether these homologs could be used in agricultural applications. We thus performed RNA-sequencing experiments with three biological replicates, comparing the cultivated soybean Williams 82 (W82) grown under control conditions (W82-Water) with W82 treated with 200 mM NaCl for 15 days (W82-NaCl). The differentially expressed genes (DEGs) were identified by a pairwise comparison of the transcriptome datasets (W82-Water vs. W82-NaCl). As expected, the gene expression patterns were similar between three biological replicates but differed significantly between the control and salt treatments (Figure 3A).

We identified 24 differentially expressed *DREB* genes in the W82-water vs. W82-NaCl comparison. There were nine up-regulated genes in the A-2 subgroup, one up-regulated gene each in the A-3 and A-5 subgroups, three up-regulated genes in the A-6 subgroup, four down-regulated genes in the A-2 and A-3 subgroups, and two down-regulated genes in the A-5 subgroup (Figures 3B,C and Supplementary Table 2).

The gene structures of the 24 differentially expressed *DREB* genes were diagrammed, including the untranslated regions (UTRs) and the locations of exons and introns (Supplementary Figure 2). Among the up-regulated *DREB* genes, nine genes had no introns and five genes contained one intron (Supplementary Figure 2A). Among the down-regulated *DREB* genes, nine genes had no introns and only one gene contained one intron (Supplementary Figure 2B). This observation is consistent with the structural characteristics described by Zhou et al. (2020b).

### The Natural Variation of *DREB3a* and *DREB3b* Affects Salt Tolerance

**FIGURE 4:**
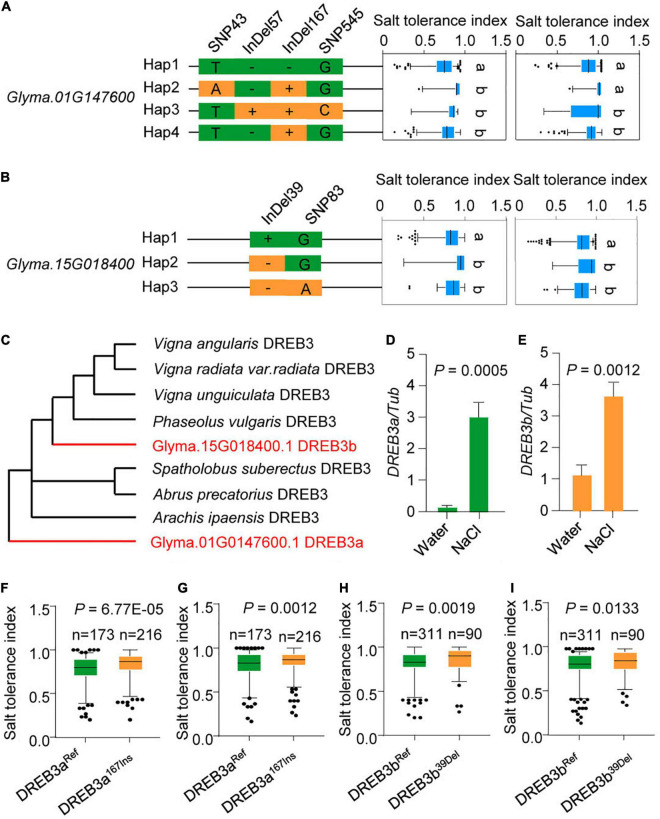
Natural variation of *DREB3a* and *DREB3b* are significantly associated with the salt tolerance. **(A)** Boxplots for salt tolerance index based on the haplotypes of *Glyma.01G147600* among 424 soybean accessions. The *Glyma.01G147600* haplotypes (Hap1–Hap4) were categorized by the four significant variants. **(B)** Boxplots for salt tolerance index based on the haplotypes of *Glyma.15G018400* among 424 soybean accessions. The *Glyma.15G018400* haplotypes (Hap1–Hap3) were categorized by the two significant variants. The experiment was performed using two biological replicates in **(A,B)**. The same lowercase letters above the histogram bars in **(A,B)** denote non-significant differences across the two panels (*P* > 0.05). One-way ANOVA was used to generate the *P*-values. **(C)** Phylogenetic tree of DREB proteins in plants. Red indicates DREB3a and DREB3b protein. **(D)** The transcription levels of *DREB3a* under salt stress (200 mM NaCl). **(E)** The transcript levels of *DREB3b* under salt stress (200 mM NaCl). All data were given as mean ± s.e.m. (*n* = 3 biological replicates). **(F,G)** *GmDREB3a^Ref^* and*DREB3a^167Ins^* alleles associated with salt tolerance index. **(H,I)** *DREB3b^Ref^* and*DREB3b^39Del^* alleles associated with salt tolerance index. One-tailed Student’s *t*-test was used to generate the *P*-values in **(D–I)**.

Some *DREB* genes positively regulate tolerance to abiotic stress in soybean (Chen et al., 2007, 2009; Zhou et al., 2020a); therefore, we focused on the 14 up-regulated *DREB* genes. To systematically study natural variation of *DREB* genes, we investigated the variation of all 14 up-regulated *DREB* genes in the 424-accession panel and found that only nine *DREB* genes arose through natural variation: *Glyma.01G147600*, *Glyma.15G018400*, *Glyma.04G084000*, *Glyma.08G035000*, *Glyma.16G023800*, *Glyma.09G233800*, *Glyma.08G137600*, *Glyma.12G103100*, *Glyma.06G301300* (Figures 4A,B and Supplementary Figures 3–6).

We then measured the salt tolerance indexes of the population to determine if salt tolerance is associated with any of the nine genes. After correcting for population structure (at least ten individual plants were analyzed per accession), two out of the nine *DREB* genes were significantly correlated with the salt tolerance index (*p* < 0.05): *Glyma.01G147600* and *Glyma.15G018400* (Figures 4A,B).

Phylogenetic investigation indicated that *Glyma.01G147600* and *Glyma.15G018400* are orthologs of *DREB3* in other plants. The two orthologs were thus named *DREB3a* and *DREB3b*, respectively (Figure 4C). To determine whether *DREB3a* and *DREB3b* respond to salt stress, we performed qRT-PCR to examine their expression profiles under salt stress. The results showed that *DREB3a* and *DREB3b* were up-regulated under salt stress (Figures 4D,E), consistent with RNA-sequencing data (Figure 3C).

Interestingly, Hap. 2-4 of *DREB3a* harbored a 15-bp InDel predicted to insert 5 amino acids, and Hap. 2–3 of *DREB3b* lacked a 6-bp InDel predicted to lack 2 amino acids (hereafter referred to as *DREB3a^167Ins^* and *DREB3b^39Del^*, respectively). We then compared the phenotypic differences of the accessions carrying either the reference genome *DREB3a* allele (*DREB3a^Ref^*) or *DREB3a^167Ins^* allele, and the two accessions carrying either the reference genome *DREB3b* allele (*DREB3b^Ref^*) or *DREB3b^39Del^* allele. The salt tolerance indexes of *DREB3a^167Ins^* and *DREB3b^39Del^* allele accessions were significantly higher than *DREB3a^Ref^* and *DREB3b^Ref^* accessions in two biological replicates (Figures 4F–I). These results strongly suggest that *DREB3a* and *DREB3b* are excellent candidate genes that respond to salt stress.

### *DREB3b*, but Not *DREB3a*, Has Undergone Artificial Selection in the Domestication Process

To gain further insight into the evolutionary history of *DREB3a* and *DREB3b*, we examined the variation in the *DREB3a* and *DREB3b* coding sequences in our previously described collection of 1295 resequenced accessions (Supplementary Figure 7A), of which four (Hap. 1, 2, 3, and 4) are described above (Figure 4A), and identified five haplotypes in *DREB3b* (Supplementary Figure 7B), of which three (Hap. 1, 2, 3) are described above (Figure 4B). The residues of Hap. 2 for both *DREB3a* and *DREB3b* are identical to all other homologs in legume (Supplementary Figures 8A,B), suggesting that Hap. 2 is the original haplotype in the soybean species. Median-joining network analysis of *DREB3a* showed that Hap. 5 is derived from Hap. 2, Hap. 1, and Hap. 3 are derived from Hap. 5, and Hap. 4 is derived from Hap. 3 (Supplementary Figure 9A). Median-joining network analysis of *DREB3b* showed that Hap. 1, 3, and 5 are derived from Hap. 2, and Hap. 4 is derived from Hap. 3 (Supplementary Figure 9B).

**FIGURE 5:**
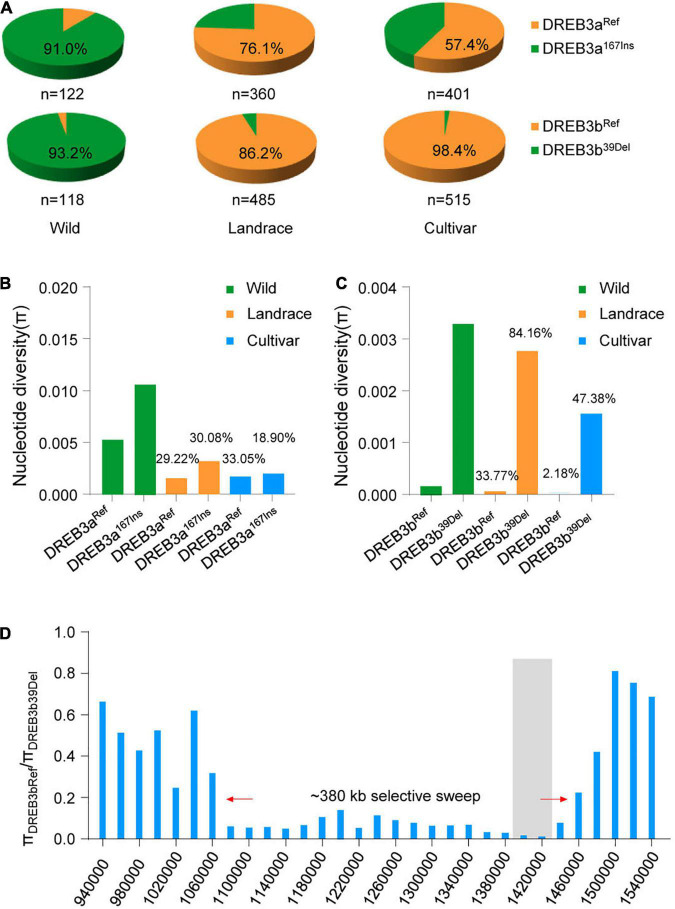
*DREB3b* underwent artificial selection but *DREB3a* did not. **(A)** Proportions of *DREB3a* and *DREB3b* alleles and their co-occurrence with each of the three germplasm groups (wild, landrace, and cultivar). **(B)** Nucleotide diversity analysis of the region surrounding *DREB3a* in wild, landrace, and cultivated soybean. **(C)** Nucleotide diversity analysis of the region surrounding *DREB3b* in wild, landrace, and cultivated soybean. **(D)** Selective sweep in the 380-kb *DREB3b* genomic region.

We then analyzed the percentage of *DREB3a^167Ins^* and *DREB3b^39Del^* alleles in 1295 resequenced soybean accessions. Surprisingly, the most common allele *DREB3b^Ref^* was present in the vast majority of improved cultivars (507/515) and in most landraces (418/485) (Figure 5A), indicating that the *DREB3b^Ref^* allele is nearly fixed in the landrace population. This dramatic increase of the *DREB3b^Ref^* allele in the soybean population indicates that the *DREB3b^Ref^* allele might have been under strong artificial selection during domestication.

To examine whether selection has acted on *DREB3b^Ref^*, we analyzed the nucleotide diversity of the 20-kb region flanking *DREB3b^Ref^* in domesticated and wild soybean lines. Landraces carrying the *DREB3b^39Del^* allele retained 84.16% of the nucleotide diversity of their wild counterparts, and improved cultivars retained 47.38% of the nucleotide diversity of landraces (Figure 5C). Whereas, landraces carrying the *DREB3b^Ref^* allele retained only 33.77% of the nucleotide diversity of wild lines, and improved cultivars retained only 2.18% of the nucleotide diversity of landraces (Figure 5C). We identified strong evidence of selection in a region of 380 kb around the *DREB3b* gene (Figure 5D). These results suggest that the *DREB3b^Ref^* allele was targeted by selection, thereby causing its rapid accumulation in domesticated soybeans. This is also supported by similar negative values of Tajima’s *D* statistic for *DREB3b* in domesticated soybeans (Supplementary Table 5), indicating that *DREB3b* (and in particular the *DREB3b^Ref^* allele) experienced selection during the transition from wild soybean to landrace.

Given that salt tolerance is not considered a soybean domestication trait, it is not clear why *DREB3b^Ref^* was selected during early domestication. We propose that the *DREB3b* region is a hotspot for many domestication and agronomic traits. Consistent with expectation, the *DREB3b^Ref^* allele improves flowering, increases seed size, and decreases plant height relative to the *DREB3b^39Del^* allele (Supplementary Figures 10A–C). Therefore, *DREB3b^Ref^* may have been selected for an effect on flowering time, seed size and plant height, and the change in salt tolerance was a correlated response. Considering the enrichment of the *DREB3b^39Del^* allele in wild soybeans, and the higher salt tolerance index of the *DREB3b^39Del^* allele compared to that of the *DREB3b^Ref^* allele, the *DREB3b^39Del^* allele may be undergoing strong natural selection to facilitate the response of wild soybeans to salt stress.

For *DREB3a*, the *DREB3a^Ref^* allele was the most abundant in landraces (274/360) and in improved cultivars (230/401) (Figure 5A). However, landraces carrying the *DREB3a^167Ins^* allele retained 30.08% of the nucleotide diversity of their wild counterparts, and improved cultivars retained 18.90% of the nucleotide diversity of landraces (Figure 5B). Whereas, landraces carrying *DREB3a^Ref^* allele retained 29.22% of the nucleotide diversity of wild, and cultivated retained 33.05% of the nucleotide diversity of landraces (Figure 5B). We identified no strong evidence of selection in a region of the *DREB3a* gene (Figure 5D, Supplementary Figure 11, and Supplementary Table 5). This implies that *DREB3b*, but not *DREB3a*, has undergone artificial selection during in the domestication process.

### *DREB3b^39Del^* Enhances Salt Tolerance in Soybean

**FIGURE 6:**
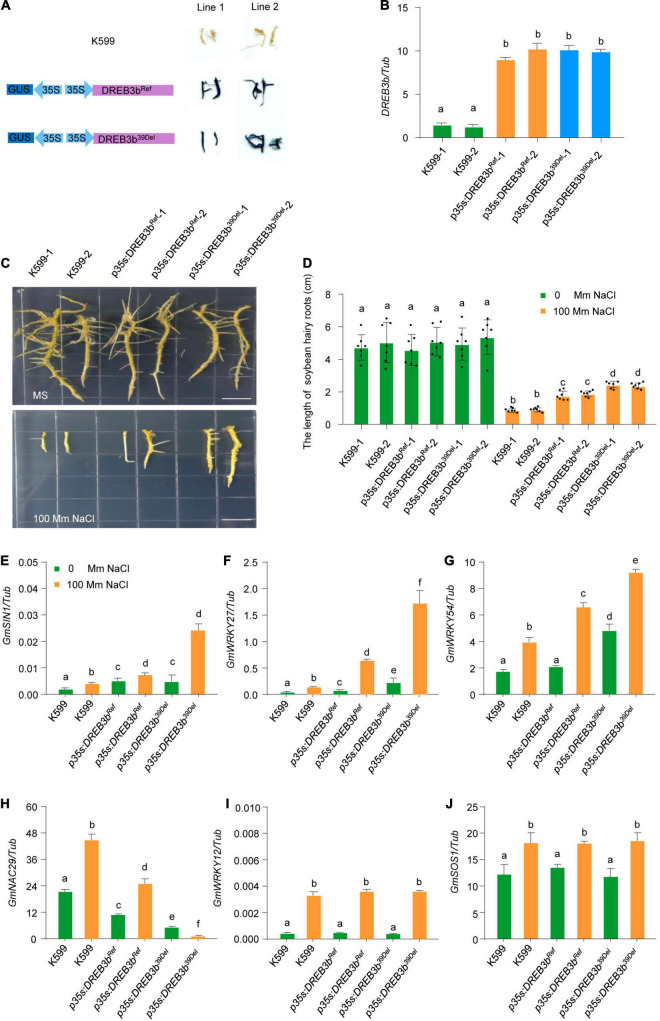
*DREB3b^39Del^* improves salt tolerance more than *DREB3b^Ref^* in transgenic soybean hairy roots. **(A)** Schematic diagram of overexpression vector and GUS staining of overexpressing *DREB3b^39Ref^* and*DREB3b^39Del^* in soybean hairy roots. **(B)** The transcript levels of *DREB3b* in W82 and two independent *DREB3b^39Ref^* and *DREB3b^39Del^* transgenic lines grown under normal growth conditions. **(C,D)** Phenotype of the transgenic lines overexpressing *DREB3b^39Ref^* and *DREB3b^39Del^* with or without 100 mM NaCl treatment. The lengths of soybean hairy roots were obtained from at least seven roots. **(E–J)** DREB3b regulates the expression of salt stress-tolerant gene in soybean with or without 100 mM NaCl treatment. Experiment was performed using three biological replicates. The same lowercase letters above the histogram bars denote non-significant differences across the two panels (*P* > 0.05). One-way ANOVA was used to generate the *P*-values.

We next focused on the function of *DREB3b*. To further characterize the potential contribution of *DREB3b* to salt tolerance in different soybean varieties, we generated *DREB3b^Ref^*-overexpressing and *DREB3b^39Del^*-overexpressing transgenic soybean hairy roots by high-efficiency *A. rhizogenes*-mediated transformation (Graham et al., 2007; Kereszt et al., 2007). We examined the *DREB3b^Ref^*-overexpressing and *DREB3b^39Del^*-overexpressing transgenic hairy roots by GUS staining and qRT-PCR (Figures 6A,B). Under normal conditions (0 mM NaCl), all soybean hairy roots grew well, with no noticeable difference (Figure 6C). Under 100 mM NaCl stress, the average root length of *DREB3b^39Del^*-overexpressing soybean hairy roots was much longer than that of *DREB3b^Ref^*-overexpressing transgenic hairy roots and much longer than that of non-transgenic hairy roots (Figures 6C,D).

Many positive and negative regulators of salt tolerance in soybean have been identified (Zhou et al., 2008; Wang et al., 2015; Shi et al., 2018; Li et al., 2019). To test whether DREB3b can regulate the expression of these genes, we performed qRT-PCR analysis in *DREB3b^Ref^*-overexpressing and *DREB3b^39Del^*-overexpressing soybean hairy roots under mock and NaCl treatment (100 mM). *DREB3b^Ref^* and *DREB3b^39Del^* up-regulated the expression of the positive regulators of salt tolerance *SIN1*, *WRKY27*, and *WRKY54*, and down-regulated the expression of negative regulator of salt tolerance *NAC29*, but could regulate the expression of *WRKY12* and *SOS1* under mock and NaCl treatment (Figures 6E–J). Interestingly, the transcript levels of *SIN1*, *WRKY27* and *WRKY54* were higher in *DREB3b^39Del^* soybean hairy roots than in *DREB3b*^Ref^** soybean hairy roots, and the transcript level of *NAC29* was lower in *DREB3b^39Del^* soybean hairy roots than in *DREB3b*^Ref^** soybean hairy roots under NaCl treatment (Figures 6E–J). These results show that the *DREB3b^39Del^* allele enhances salt tolerance more than the *DREB3b^Ref^* allele in soybean.

Genetic markers are an important and effective tool for identifying mutant alleles in molecular-assisted studies, and could accelerate the genotyping procedure in future generations. We thus developed an InDel marker to identify the *DREB3b^39Del^* allele. After PCR amplification using *DREB3b*-specific primers (Supplementary Table 3), the *DREB3b^Ref^* allele produced 106-bp amplicons, whereas the *DREB3b^39Del^* allele produced 100-bp amplicons, and the differences could be detected by electrophoresis (Supplementary Figure 12). We used this InDel marker to detect the genotypes of soybean accessions at random, and the results were consistent with the sequencing results (Supplementary Figure 12 and Supplementary Table 4). Therefore, this InDel marker can be used to improve the breeding efficiency of salt tolerant soybean varieties in molecular breeding.

## Discussion

Soybean is a leguminous plant that is capable of closed flower fertilization (closed flower pollination). This feature may be helpful for maintaining genomic homogeneity and reducing genomic variation, which may have been further intensified by the domestication process (Lam et al., 2010). Due to bottlenecks and artificial selection, some genes that play important roles for adaptation to different environments have been lost during the soybean domestication process. Wild soybean (*Glycine soja*) may have high allelic diversity at certain loci, including advantageous alleles that can be re-introduced into domesticated soybeans via breeding to improve their adaption to the environment (Zhang et al., 2017). Consistent with a previous report (Lam et al., 2010), we show that the SNPs number of both landrace and improved cultivars were significantly lower than that of wild soybeans (Figure 1B), and wild soybeans have retained higher genetic diversity, but have been lost in cultivated soybeans (Figure 1C). We also show that the salt tolerance index of wild soybeans was much higher than that of improved cultivars (Figures 1D,E). These data indicate that a large number of useful alleles and genes from wild soybeans may be great significance for improving the salt tolerance of soybean. Importantly, to explore the variation in excellent alleles in wild soybeans, we combined genome-wide analysis and RNA-sequencing and identified an excellent allele from wild soybean, which could be useful in breeding for improved salt tolerance in soybean.

Farmlands worldwide are increasingly affected by soil salinization, and most crops are sensitive to salt stress (Ren et al., 2005; Munns et al., 2012; An et al., 2017). Therefore, salt tolerance is an ecologically significant trait for crop breeding. Previous studies have confirmed that genetic variation in salt tolerance exists widely within and among major crops, providing an opportunity to use salt tolerance variation to improve crop salt tolerance (Wu et al., 2012; Qi et al., 2014; Zhang et al., 2018). For example, natural variation in *SALT3*/*CHX1*, a cation H^+^ exchanger gene, could modulate salt tolerance in soybean (Guan et al., 2014; Qi et al., 2014). Moreover, the DREB family is one of the most important TF families in vascular plants, and DREB TFs are involved in salt stress responses (Li et al., 2005; Xu et al., 2011; Kudo et al., 2017; Ali and Hadi, 2018; Eckardt, 2019). For example, *DREBa*, *DREBb*, and *DREBc* are up-regulated under salt stress conditions (Li et al., 2005); GmDREB2, a soybean DRE-binding transcription factor, confers drought- and salt-tolerance in transgenic Arabidopsis and tobacco (*Nicotiana tabacum*) plants (Chen et al., 2007). However, the natural variation of *DREB* genes has not been examined in soybean. Here, we have identified one *DREB* gene in soybean, *DREB3b*, which conferred salt tolerance in soybean (Figure 4B). The molecular footprint showed strong selection at *DREB3b* during domestication (Figure 5D). The *DREB3b^39Del^* allele was highly abundant in wild soybeans (about 95%), and the *DREB3b^Ref^* allele was highly abundant in improve cultivar (about 98%, Figure 5A). Together, the data suggest that the DREB3bRef allele may have undergone artificial selection during domestication. We also demonstrate that, compared to *DREB3b^Ref^* overexpression, *DREB3b^39Del^* overexpression led to (i) better salt tolerance in soybean hairy root, and (ii) stronger up-regulation of positive regulators of salt tolerance and stronger down-regulation of a negative regulator of salt tolerance. Therefore, the loss of the *DREB3b^39Del^* allele in the domestication of improved cultivar may be a reason for its reduced salt tolerance.

**FIGURE 7:**
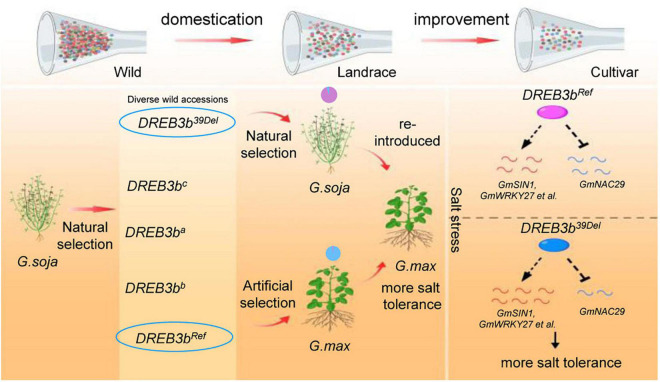
Model of artificial selection in *DREB3b* in soybean and the underlying mechanism. A large number of potentially advantageous alleles were lost during domestication and cultivar improvement, resulting in lower salt tolerance of cultivated soybean than wild soybean. The *DREB3b^Ref^* allele underwent artificial selection in cultivated soybean. The *DREB3b^Ref^* allele reduced the salt tolerance of cultivated soybean. DREB3b up-regulates positive regulators of salt tolerance and down-regulates negative regulators of salt tolerance.

Based on our data, we propose a model (Figure 7) describing how the artificial selection of the *DREB3b^Ref^* allele occurred in cultivated soybean, thereby reducing salt tolerance. According to this model, wild soybeans contain a large number of alleles in *DREB3b* but the *DREB3b^Ref^* allele underwent artificial selection in the domestication of cultivated soybean, resulting in the reduction of salt tolerance. Overexpression of *DREB3b* could improve salt tolerance by up-regulating positive regulators of salt tolerance and down-regulating negative regulators of salt tolerance.

## Data Availability Statement

The datasets presented in this study can be found in online repositories. The names of the repository/repositories and accession number(s) can be found at: NCBI BioProject, PRJNA797227.

## Author Contributions

QC and BL designed and interpreted the results. QC, BL, DZ, and NF coordinated the projects. QC, LD, FK, HD, TL, YC, YL, and ZH performed the experiments. LD, QC, YL, and ZH performed the data analysis. QC and ZH wrote the manuscript. WL revised the manuscript. All authors contributed to the article and approved the submitted version.

## Conflict of Interest

YC is employed by Beijing Zhongnong Futong Horticulture Co., Ltd. The remaining authors declare that the research was conducted in the absence of any commercial or financial relationships that could be construed as a potential conflict of interest.

## Publisher’s Note

All claims expressed in this article are solely those of the authors and do not necessarily represent those of their affiliated organizations, or those of the publisher, the editors and the reviewers. Any product that may be evaluated in this article, or claim that may be made by its manufacturer, is not guaranteed or endorsed by the publisher.

## Footnotes

1
https://phytozome.jgi.doe.gov/pz/portal.html

2
http://venanciogroup.uenf.br/cgi-bin/gmax_atlas/index.cgi

3
https://phytozome-next.jgi.doe.gov/

## Supplementary Material

Supplementary Figure 1 Distribution of 103 *DREB* genes on 20 chromosomes in soybean.

Supplementary Figure 2 Gene structures of 24 *DREB* genes. **(A)** Gene structures of up-regulated *DREB* genes. **(B)** Gene structures of down-regulated *DREB* genes. The exon-intron organization and UTRs of *DREB* genes were analyzed by aligning the DNA sequences using the GSDS 2.0 server (http://gsds.gao-lab.org/index.php). Introns and exons are shown as black lines and orange boxes, respectively. Green boxes at 5’ and 3’ ends represent untranslated regions (UTRs).

Supplementary Figure 3 Boxplots for salt tolerance index based on the haplotypes of salt-induced *DREB* genes from the A-2 subgroup. The experiment was performed using two biological replicates. The same lowercase letters above the histogram bars denote non-significant differences across the two panels (*P* > 0.05). One-way ANOVA was used to generate the *P*-values.

Supplementary Figure 4 Boxplots for salt tolerance index based on the haplotypes of salt-induced *DREB* gene from the A-3 subgroup. The experiment was performed using two biological replicates. The same lowercase letters above the histogram bars denote non-significant differences across the two panels (*P* > 0.05). One-way ANOVA was used to generate the *P*-values.

Supplementary Figure 5 Boxplots for salt tolerance index based on the haplotypes of salt-induced *DREB* gene from the A-5 subgroup. The experiment was performed using two biological replicates. The same lowercase letters above the histogram bars denote non-significant differences across the two panels (*P* > 0.05). One-way ANOVA was used to generate the *P*-values.

Supplementary Figure 6 Boxplots for salt tolerance index based on the haplotypes of salt-induced *DREB* genes from the A-6 subgroup. The experiment was performed using two biological replicates. The same lowercase letters above the histogram bars denote non-significant differences across the two panels (*P* > 0.05). One-way ANOVA was used to generate the *P*-values.

Supplementary Figure 7 Haplotypes of *DREB3a* and *DREB3b*. **(A)** Haplotypes of *DREB3a.* **(B)** Haplotypes of *DREB3b.* Haplotype was extracted from the 1,295 panel of 146 wild soybeans, 575 landraces and 574 improved cultivars.

Supplementary Figure 8 Comparison of the amino acid sequences of DREB3a, DREB3b and its homologs in plants. **(A)** Protein sequence comparisons of DREB3a alleles and its homologs in plants. **(B)** Protein sequence comparisons of DREB3b alleles and its homologs in plants.

Supplementary Figure 9 Haplotype origins of *DREB3a* and *DREB3b*.

Supplementary Figure 10 Boxplots for yield-related traits based on the haplotypes of *DREB3b* gene. **(A)** Flowering time. **(B)** Plant height. **(C)** 100-grain weight. One-tailed Student’s *t*-test was used to generate the *P*-values.

Supplementary Figure 11 Selective sweep in the *DREB3a* genomic region.

Supplementary Figure 12 Development of functional genetic markers for *DREB3b^Ref^* and *DREB3b^39Del^* alleles. Lines 1–7, soybean plants harbor DREB3bRef allele. Lines 8–13, soybean plants harbor DREB3bRef allele.
