# Supplementary figures and images for: Genome-Wide Analysis of DREB Genes Identifies a Novel Salt Tolerance Gene in Wild Soybean (Glycine soja)
Source: Front Plant Sci. 2022 Mar 4;13:821647. doi: 10.3389/fpls.2022.821647 (PMC8931524; doi:10.3389/fpls.2022.821647)

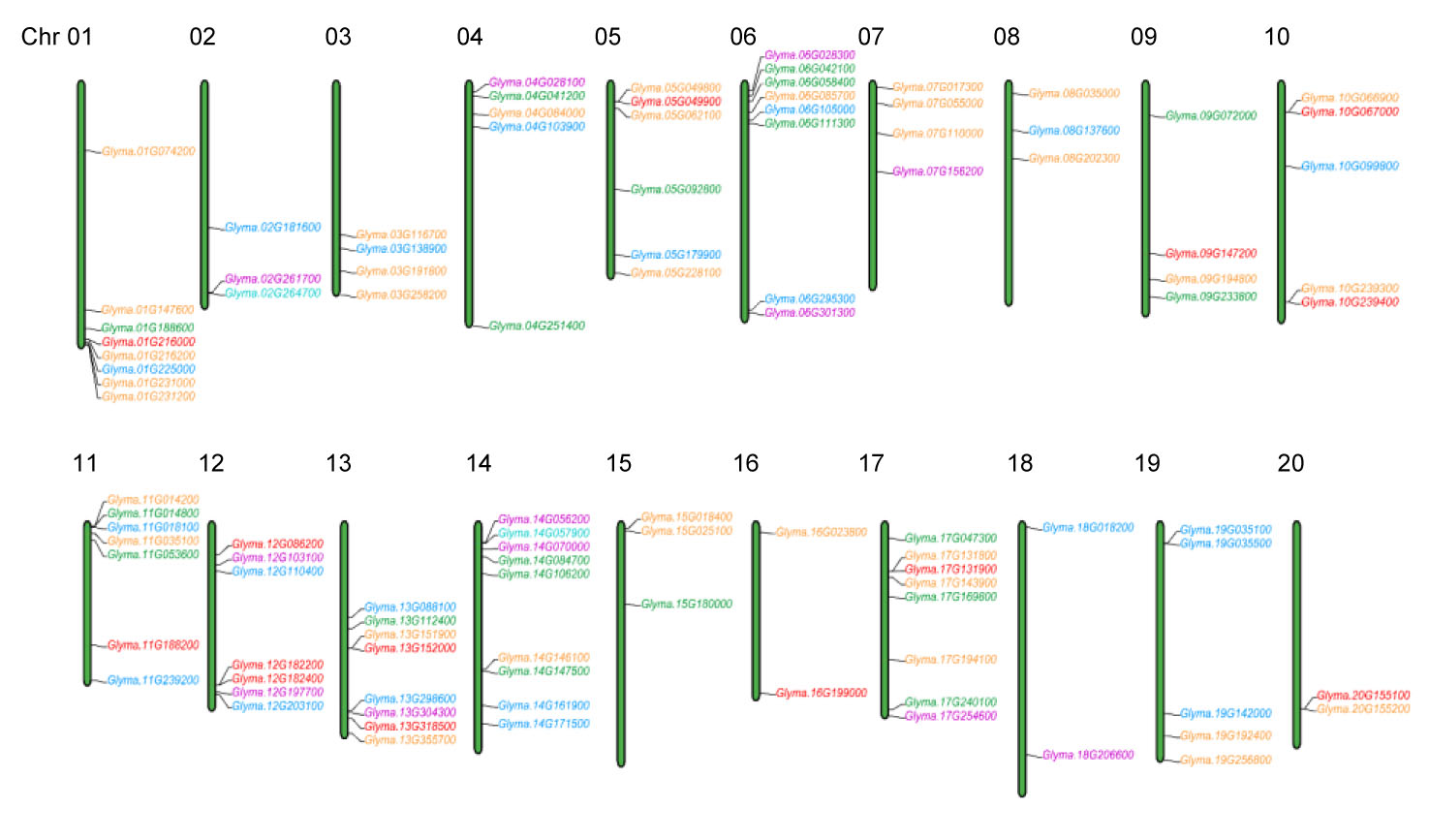

Supplement: Supplementary Figure 1 — Distribution of 103 DREB genes on 20 chromosomes in soybean. [file Image_1.JPEG]

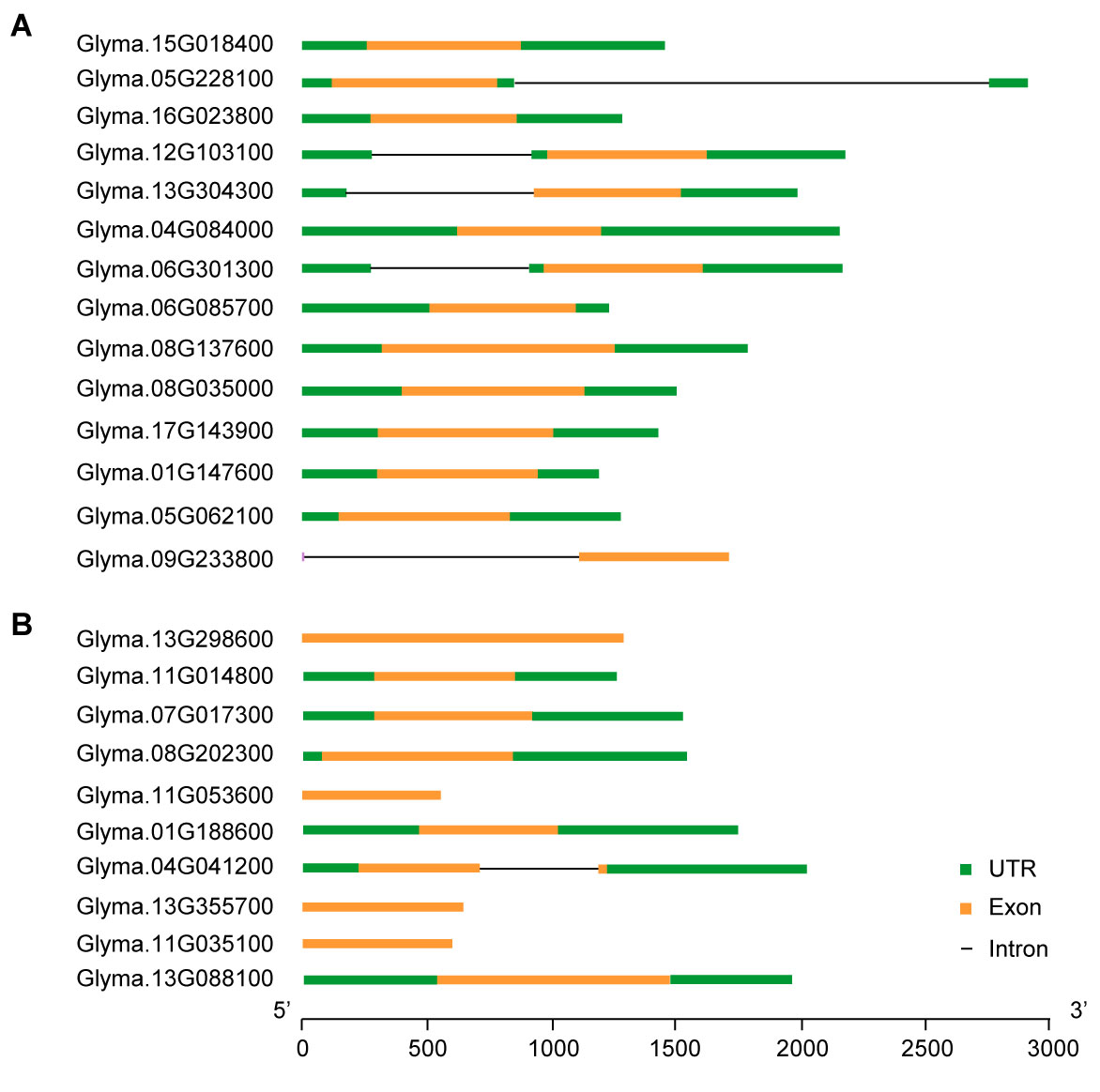

Supplement: Supplementary Figure 2 — Gene structures of 24 DREB genes. (A) Gene structures of up-regulated DREB genes. (B) Gene structures of down-regulated DREB genes. The exon-intron organization and UTRs of DREB genes were analyzed by aligning the DNA sequences using the GSDS 2.0 server (http://gsds.gao-lab.org/index.php). Introns and exons are shown as black lines and orange boxes, respectively. Green boxes at 5’ and 3’ ends represent untranslated regions (UTRs). [file Image_2.JPEG]

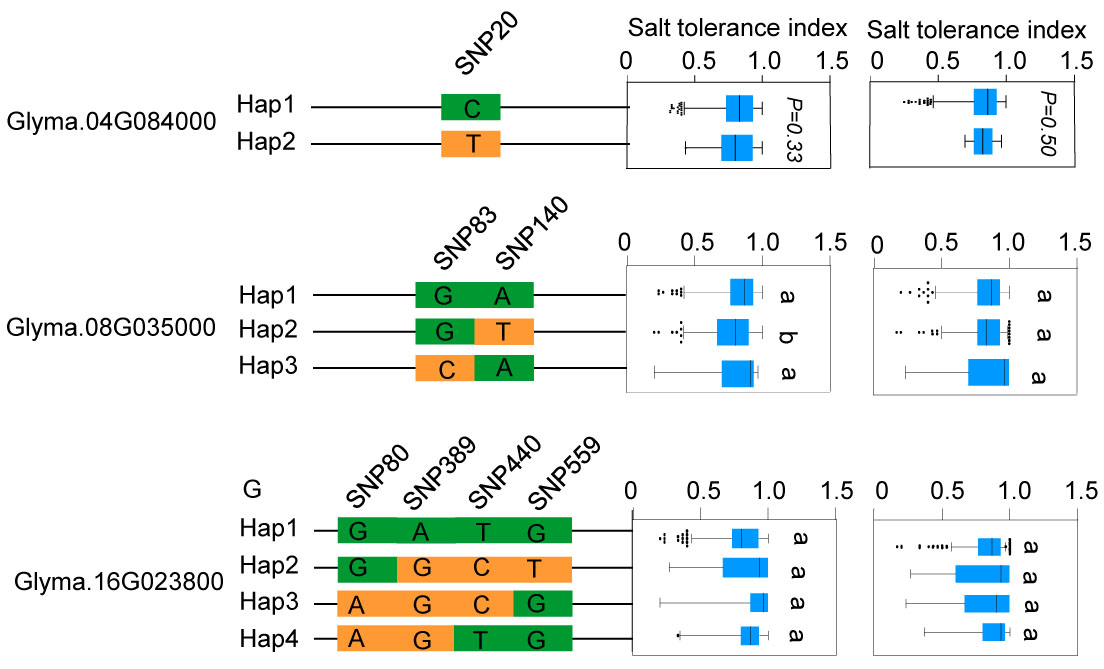

Supplement: Supplementary Figure 3 — Boxplots for salt tolerance index based on the haplotypes of salt-induced DREB genes from the A-2 subgroup. The experiment was performed using two biological replicates. The same lowercase letters above the histogram bars denote non-significant differences across the two panels (P > 0.05). One-way ANOVA was used to generate the P-values. [file Image_3.JPEG]

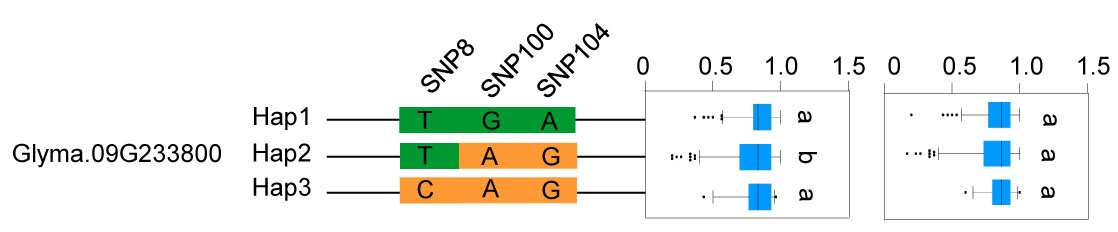

Supplement: Supplementary Figure 4 — Boxplots for salt tolerance index based on the haplotypes of salt-induced DREB gene from the A-3 subgroup. The experiment was performed using two biological replicates. The same lowercase letters above the histogram bars denote non-significant differences across the two panels (P > 0.05). One-way ANOVA was used to generate the P-values. [file Image_4.JPEG]

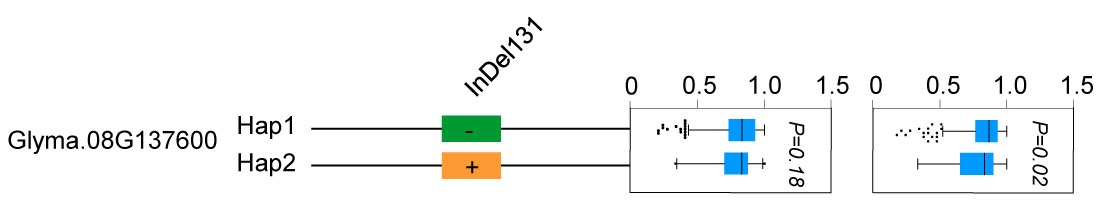

Supplement: Supplementary Figure 5 — Boxplots for salt tolerance index based on the haplotypes of salt-induced DREB gene from the A-5 subgroup. The experiment was performed using two biological replicates. The same lowercase letters above the histogram bars denote non-significant differences across the two panels (P > 0.05). One-way ANOVA was used to generate the P-values. [file Image_5.JPEG]

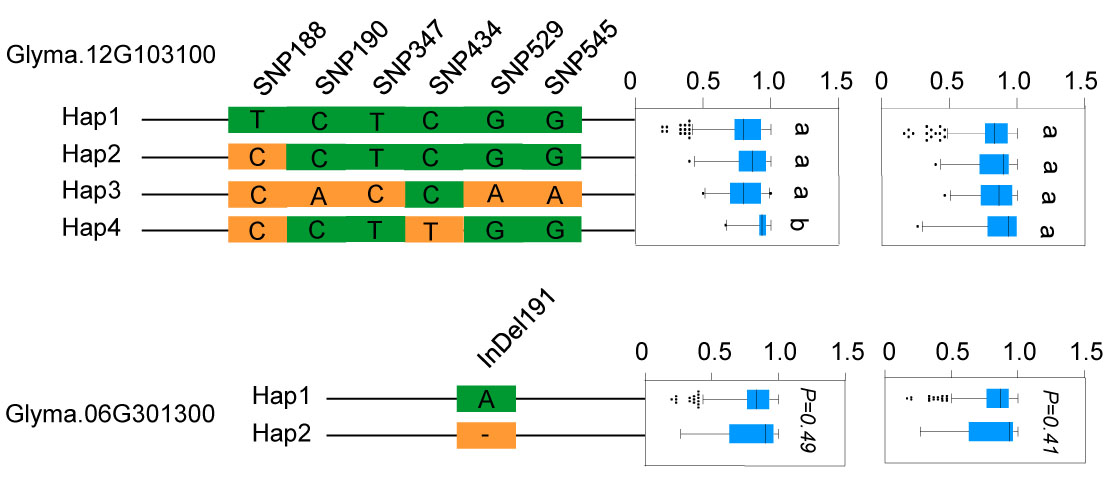

Supplement: Supplementary Figure 6 — Boxplots for salt tolerance index based on the haplotypes of salt-induced DREB genes from the A-6 subgroup. The experiment was performed using two biological replicates. The same lowercase letters above the histogram bars denote non-significant differences across the two panels (P > 0.05). One-way ANOVA was used to generate the P-values. [file Image_6.JPEG]

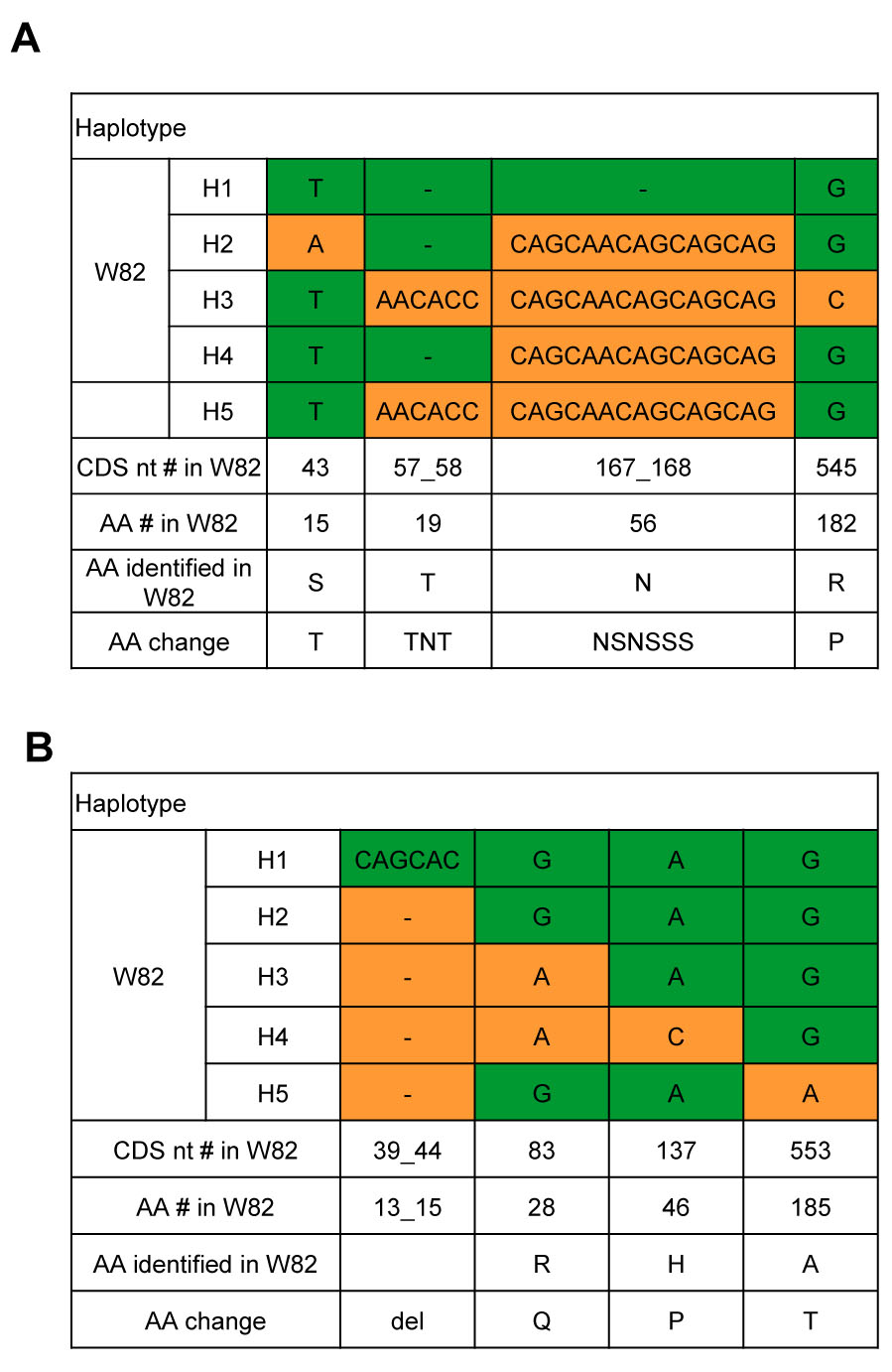

Supplement: Supplementary Figure 7 — Haplotypes of DREB3a and DREB3b. (A) Haplotypes of DREB3a. (B) Haplotypes of DREB3b. Haplotype was extracted from the 1,295 panel of 146 wild soybeans, 575 landraces and 574 improved cultivars. [file Image_7.JPEG]

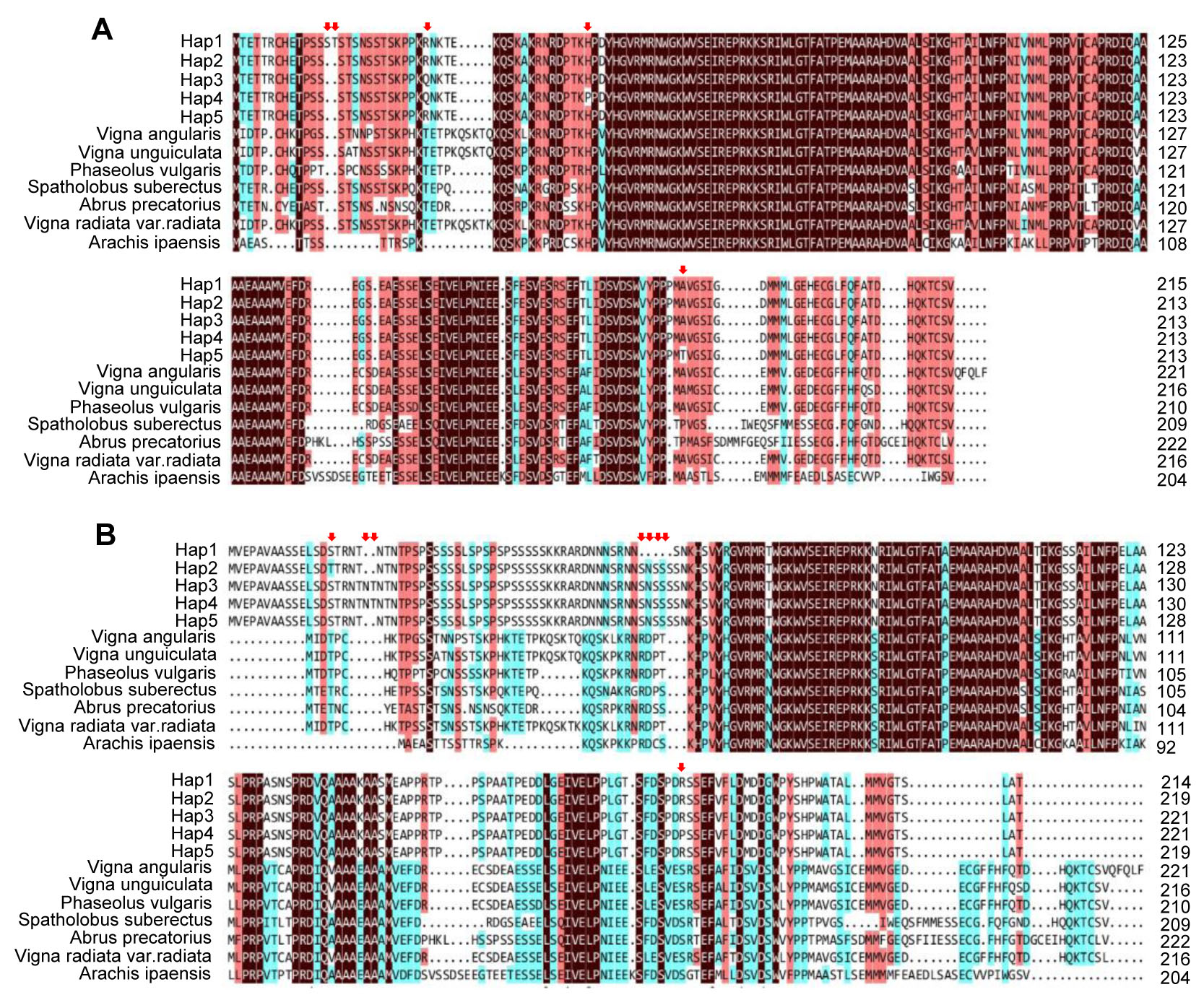

Supplement: Supplementary Figure 8 — Comparison of the amino acid sequences of DREB3a, DREB3b and its homologs in plants. (A) Protein sequence comparisons of DREB3a alleles and its homologs in plants. (B) Protein sequence comparisons of DREB3b alleles and its homologs in plants. [file Image_8.JPEG]

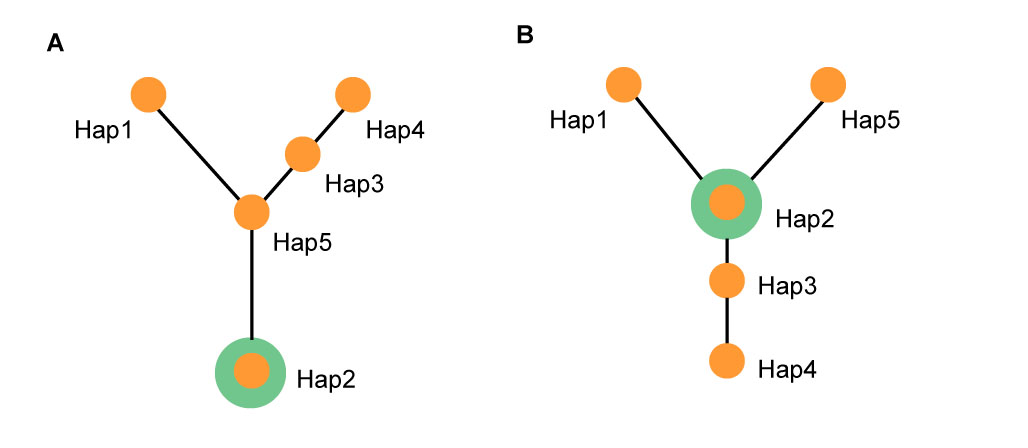

Supplement: Supplementary Figure 9 — Haplotype origins of DREB3a and DREB3b. [file Image_9.JPEG]

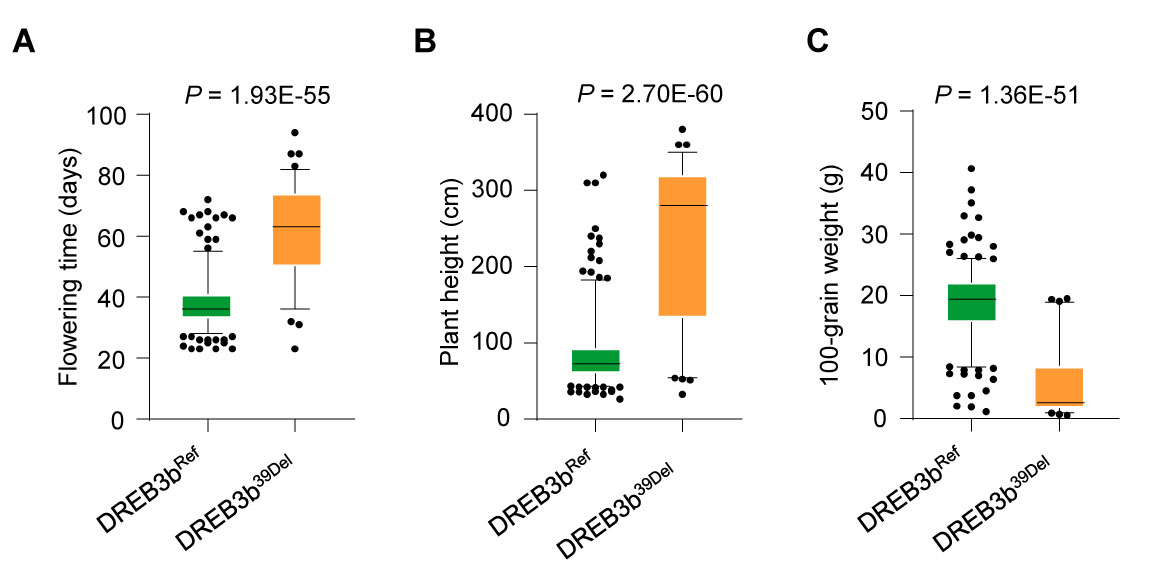

Supplement: Supplementary Figure 10 — Boxplots for yield-related traits based on the haplotypes of DREB3b gene. (A) Flowering time. (B) Plant height. (C) 100-grain weight. One-tailed Student’s t-test was used to generate the P-values. [file Image_10.JPEG]

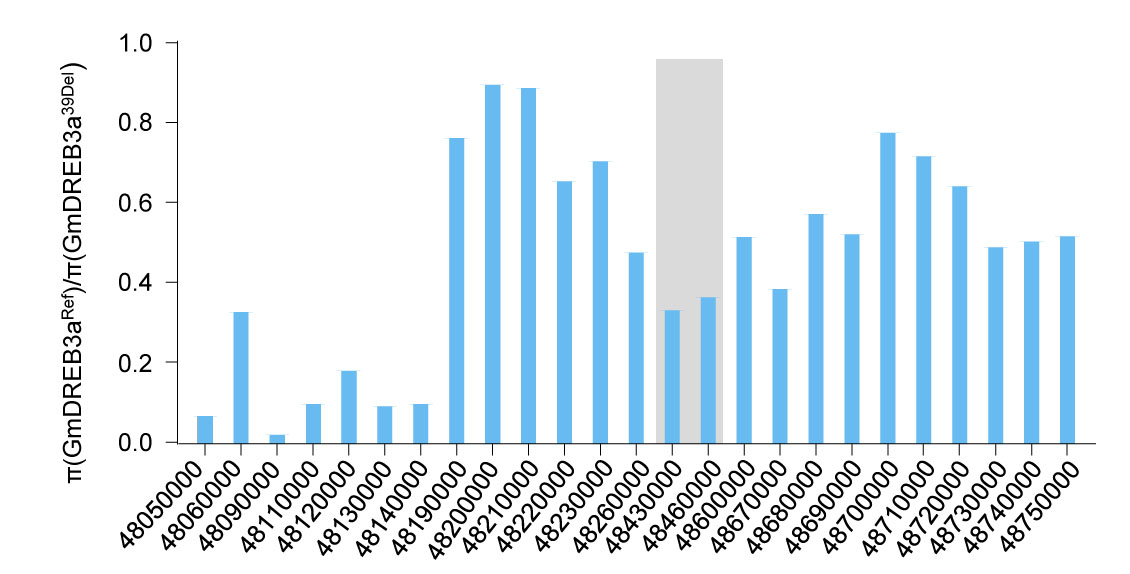

Supplement: Supplementary Figure 11 — Selective sweep in the DREB3a genomic region. [file Image_11.JPEG]

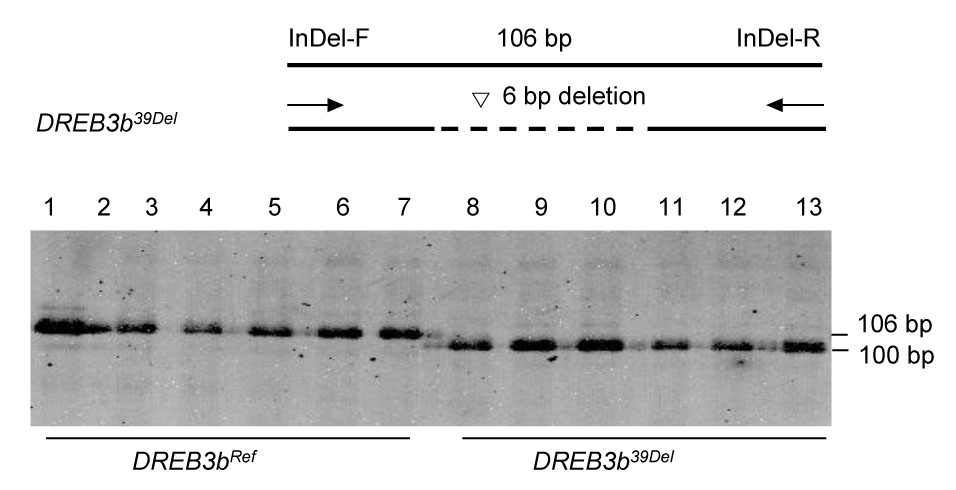

Supplement: Supplementary Figure 12 — Development of functional genetic markers for DREB3bRef and DREB3b39Del alleles. Lines 1–7, soybean plants harbor DREB3bRef allele. Lines 8–13, soybean plants harbor DREB3bRef allele. [file Image_12.JPEG]
